# A comprehensive characterization of simple sequence repeats in pepper genomes provides valuable resources for marker development in *Capsicum*

**DOI:** 10.1038/srep18919

**Published:** 2016-01-07

**Authors:** Jiaowen Cheng, Zicheng Zhao, Bo Li, Cheng Qin, Zhiming Wu, Diana L. Trejo-Saavedra, Xirong Luo, Junjie Cui, Rafael F. Rivera-Bustamante, Shuaicheng Li, Kailin Hu

**Affiliations:** 1 College of Horticulture, South China Agricultural University, Guangzhou 510642, China; 2 Department of Computer Science, City University of Hong Kong, Hong Kong 999077, China; 3 Pepper Institute, Zunyi Academy of Agricultural Sciences, Zunyi, Guizhou 563102, China; 4 College of Horticulture and Landscape Architecture, Zhongkai University of Agriculture and Engineering, Guangzhou 510225, China; 5 Departamento de Ingeniería Genética, Centro de Investigación y de Estudios Avanzados del IPN (Cinvestav)-Unidad Irapuato, Irapuato 36821, México

## Abstract

The sequences of the full set of pepper genomes including nuclear, mitochondrial and chloroplast are now available for use. However, the overall of simple sequence repeats (SSR) distribution in these genomes and their practical implications for molecular marker development in *Capsicum* have not yet been described. Here, an average of 868,047.50, 45.50 and 30.00 SSR loci were identified in the nuclear, mitochondrial and chloroplast genomes of pepper, respectively. Subsequently, systematic comparisons of various species, genome types, motif lengths, repeat numbers and classified types were executed and discussed. In addition, a local database composed of 113,500 *in silico* unique SSR primer pairs was built using a homemade bioinformatics workflow. As a pilot study, 65 polymorphic markers were validated among a wide collection of 21 *Capsicum* genotypes with allele number and polymorphic information content value per marker raging from 2 to 6 and 0.05 to 0.64, respectively. Finally, a comparison of the clustering results with those of a previous study indicated the usability of the newly developed SSR markers. In summary, this first report on the comprehensive characterization of SSR motifs in pepper genomes and the very large set of SSR primer pairs will benefit various genetic studies in *Capsicum*.

Simple sequence repeats (SSRs), also termed microsatellites, consist of tandemly arranged repeats of short DNA motifs (1–6 bp in length). Since they were first documented in the 1980s^1^ and their abundance and ubiquity has been confirmed in prokaryotic and eukaryotic genomes in subsequent studies^2^,^3^, SSRs have become one of the most attractive markers for plant genetics and breeding^4^. As molecular markers, SSRs present several important advantages, such as being locus-specific and multi-allelic, exhibiting co-dominant transmission, their ease of detection by PCR and their high rates of transferability across species^5^. SSRs have been extensively involved in a variety of applications including cultivar identification^6^, the determination of ‘hybridity’^7^, genetic diversity assessment^8^, genetic mapping^9^, gene tagging^10^, gene flow^11^ and molecular evolution^12^ in various plant and animal systems.

In general, SSR markers for plant studies have been discovered by cross-species amplification^13^, screening either SSR-enriched cDNA or genomic libraries^14^ and searching public databases^15^. With the decreasing cost of next generation sequencing (NGS), which is a powerful and convenient tool for marker discovery^16^, SSRs can now be identified on a large scale from the *de novo* assembled transcriptome or whole genome^17^. In the latter case, the whole picture of SSR frequency and distribution can be concurrently described^18^ and provide practical implications for their use as molecular markers^19^. Genome-wide SSR identification has been performed in many organisms including humans^20^, marine animals^21^, insects^22^, medicinal fungi^23^ and certain economically valuable plants^24^,^25^,^26^. However, except for cucumber^27^ and Chinese cabbage^28^, the effort in this area for important vegetable species such as *Capsicum* spp. lags far behind.

In addition, similar to the nuclear genomes, SSRs in organelle genomes are common^29^ and it is generally accepted that polymorphisms due to variations in SSR motif length in the chloroplast or mitochondrial genomes would also be of considerable practical value for monitoring gene flow^30^, population differentiation^31^ and cytoplasmic diversity^32^. To our knowledge, a complete analysis of SSR loci in the mitochondrial or chloroplast genomes has been performed only in a relatively limited set of species, such as bryophytes^33^, rice^34^ and soybean^29^. Thus far, this analysis has rarely been fully conducted in horticultural crops including pepper.

Pepper (*Capsicum* spp.) belongs to the Solanaceae family and is one of the most economically important vegetable crops with versatile applications for food, spice, ornamental and medicinal purposes^35^. A variety of marker systems, such as restriction fragment length polymorphisms (RFLPs), amplified fragment length polymorphisms (AFLPs), random amplified polymorphic DNA (RAPDs), single nucleotide polymorphisms (SNPs), insertion/deletion (InDel) polymorphisms and SSRs have been adopted in pepper molecular genetics research^35^,^36^,^37^,^38^,^39^,^40^,^41^,^42^,^43^,^44^,^45^,^46^. However, the total number of publicly available PCR-based anchored markers, including SSR markers, is still insufficient^46^,^47^.

In addition, the development of SSR markers of pepper in previous studies mainly focused on mining from either selected SSR-enriched libraries^39^,^48^,^49^ or public database^50^,^51^,^52^. The massive amount of RNA-seq data has facilitated the *in silico* generation of SSR information with unprecedented dimensions^53^,^54^,^55^,^56^,^57^,^58^. Nevertheless, a systematic survey of SSRs in the pepper, including nuclear, mitochondrial and chloroplast genomes, has not yet been conducted despite the recent availability of the relevant information^35^,^59^,^60^,^61^,^62^. Furthermore, the utilization of these SSR loci to develop molecular markers for genetic applications such as diversity assessment, positional cloning, genome assembly and breeding activities such as marker-assisted selection (MAS) will increase continuously^63^,^64^,^65^.

Consequently, the aim of this study was to perform a genome-wide identification of SSRs in the pepper and evaluate them for marker development. We initially detected all SSR motifs in a total of six pepper genomes including nuclear, mitochondrial and chloroplast genomes, from two independent sources. Simultaneously, for comparison purposes, we performed the same analysis for another five species: tomato (*Solanum lycopersicum*), potato (*Solanum tuberosum*), cucumber (*Cucumis sativus*), Arabidopsis (*Arabidopsis thaliana*) and rice (*Oryza sativa*). Then, a comprehensive characterization and comparison of SSR frequency and distribution within different genomes were performed, and a large number of unique SSR loci were identified using bioinformatics. Finally, a collection of 21 pepper genotypes with extensive representations was selected to verify the availability of the primer pairs of those unique SSR loci. The information and primer pairs for the very large set of SSRs distributed throughout the genome would benefit pepper research and the breeding community in the future.

## Results and Discussion

### Content of SSR motifs in the pepper nuclear genome and its comparison with related plant species

**Figure 1:**
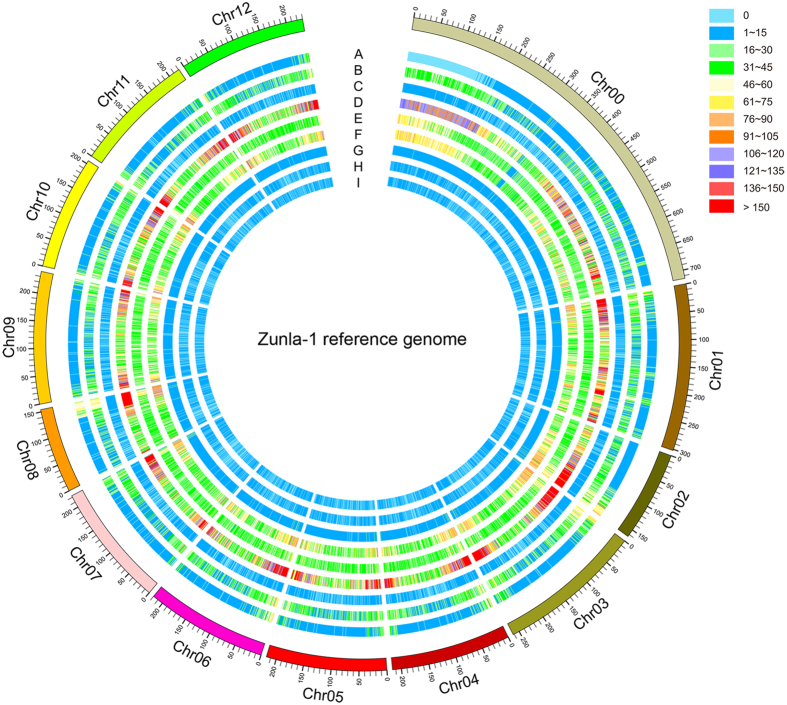
Overview of SSR distribution in the Zunla-1 reference genome. A total of 876,580 SSR loci with 136,857 present in compound formation (C and C*) that form into 739,723 SSR units were identified in the Zunla-1 reference genome. The various numbers of SSR units and protein coding genes in each window size of 1000 kb were used for drawing this picture and are shown with different colours. Track A shows the gene density; tracks B to I refer to the C, C*, Mono-, Di-, Tri-, Tetra-, Penta-, Hexa- types, respectively.

In this study, a total of 11 genomes (~9.18 Gb of sequence in total) from various species and types were collected from different databases and submitted for SSR motif identification (Supplementary Table S2). To our knowledge, unlike for *Arabidopsis*^66^, rice^67^ and cucumber^27^, there are no reports on the characterization of SSR motifs on the genome-wide level for pepper, tomato and potato. Here, the overall content of SSR motifs (1–6 bp) wit ≥4 repeats and a minimum of 10 bp length in all of the above genomic sequences was recorded (Table 1). Based on our search criteria, a total of 876,580 and 859,515 SSR loci with 136,857 and 123,281 presented in compound formation were identified in the whole genomes of Zunla-1 (N1) and Chiltepin (N2), respectively. The holistic view of their distribution patterns in the Zunla-1 and Chiltepin nuclear genomes is shown in Fig. 1 and Supplementary Figure S1, respectively. The number of SSRs identified in pepper nuclear genomes (average is 868,047.50) was approximately four times higher than the numbers found for other two Solanaceae species, tomato (235,398) and potato (241,570). If we consider into account all six species, the number of identified SSR motifs is significantly associated with genome size (R^2^ = 0.996, *P* < 0.01). However, despite possessing the largest genome size, pepper values for SSR density (SSRs/Mb) were the lowest (260.58 and 243.62 for N1 and N2, respectively) when compared to the other five species, although the two Solanaceae species, tomato and potato, showed comparable values (285.70 and 312.50 SSRs/Mb, respectively) (Table 1). In contrast, the highest density was found in cucumber (654.55 SSRs/Mb), followed by *Arabidopsis* (529.15 SSRs/Mb) and rice (527.62 SSRs/Mb). A similarly high SSR density was reported for another cucumber (Gy14) genome^27^. Nevertheless, the results from this study agreed with the trend that high SSR density is often observed in small genomes^19^. In addition, the cumulative sequence length of pepper SSR loci was approximately 16.81 Mbp and 15.90 Mbp, accounting for 0.50% and 0.45% of the assembled genomes of Zunla-1 and Chiltepin, respectively. This ratio was similar in tomato (0.59%) and potato (0.61%) but was higher than 1% in both cucumber (1.32%) and rice (1.12%) (Table 1).

### Distribution of SSR motifs in pepper organelle genomes

**Figure 2:**
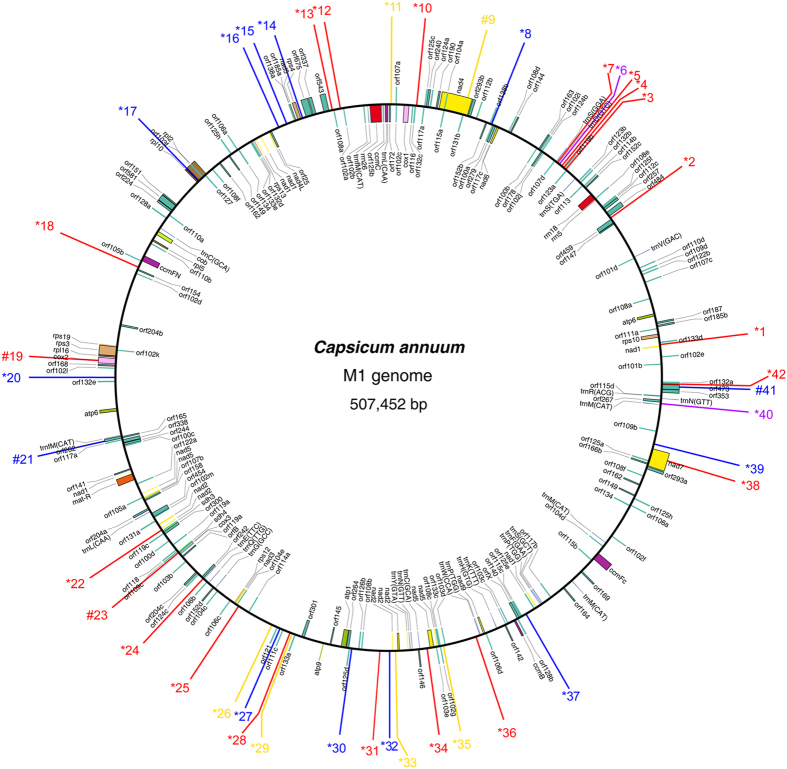
Overview of SSR distribution in pepper M1 mitochondrial genome. The M1 genome refers to the mitochondrial genome of pepper line ‘FS4401’ (*Capsicum annuum*). Perfect SSRs with 1, 2, 3 and 4 bp length of motif are represented by red, yellow, blue and green lines, respectively. Compound SSRs are shown with purple lines. Numbers with * and # in the front means that the corresponding SSRs were located in the intergenic and genic region, respectively.

**Figure 3:**
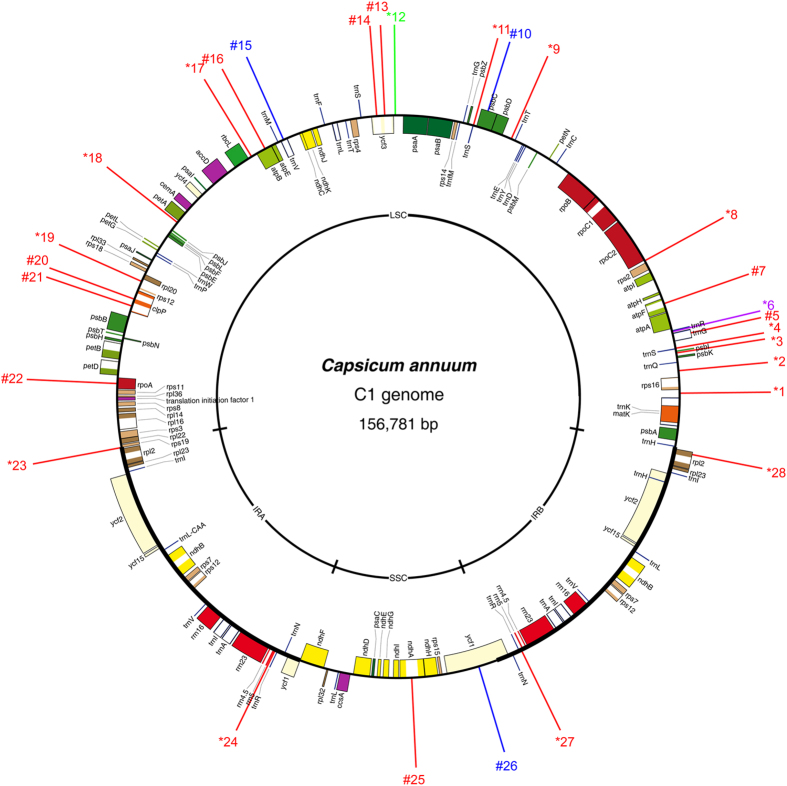
Overview of SSR distribution in pepper C1 chloroplast genome. The C1 genome refers to the chloroplast genome of the pepper line ‘FS4401’ (*Capsicum annuum*). Perfect SSRs with 1, 3 and 4 bp length of motif are represented by red, blue and green lines, respectively. Compound SSRs are shown with purple lines. Numbers with * and # in the front means that the corresponding SSRs were located in the intergenic and genic region, respectively.

**Figure 4:**
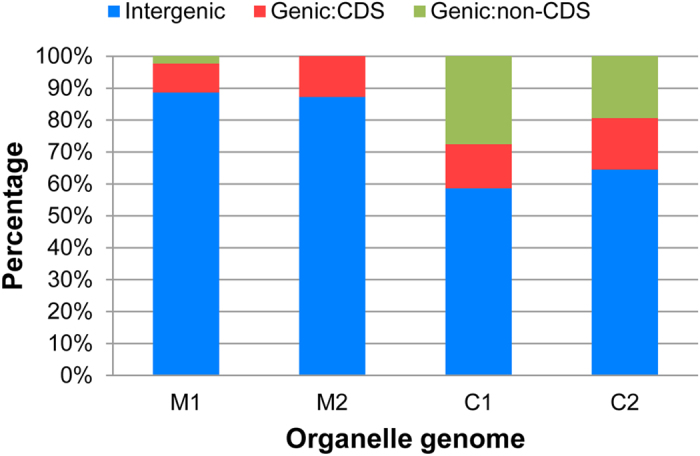
The relative proportion of SSR motifs in organelle genomes with different locations.

Similar to the results for the nuclear genomes, SSR motifs also have a wide range of distribution in the organelle genomes of various species, and they have become valuable resources for monitoring genetic flow and genetic diversity assessment at the cytoplasmic level^31^,^32^. Thus, to characterize the SSR distribution in pepper non-nuclear genomes, a total of four pepper organelle genomes were downloaded and analysed for SSR motifs. The analysis identified a total of 91 and 60 SSRs for pepper mitochondrial and chloroplast genomes, respectively (Table 1). The number of SSR loci found in each type of organelle genome was similar: 44 (M1) and 47 (M2) for the mitochondrial genomes (average 45.50) and 29 (C1) and 31 (C2) for chloroplast genomes (average 30.00) However, the overall density of SSRs in mitochondrial genomes was significantly lower than that of the pepper chloroplast (*P* = 0.000) and all the nuclear genomes (*P* = 0.001). In addition, the distribution of the mitochondrial SSRs (mtSSRs) across the genome was more even than what was observed for the chloroplast (hereafter called cpSSRs). Nevertheless, clustering of SSRs was also observed (Figs 2 and 3, Supplementary Figures S2 and S3). Notably, SSRs predominate in the long single copy (LSC) region of the chloroplast (Fig. 3 and Supplementary Figure S3). In addition, the pattern of SSR distribution in the C1 genome showed very high comparability with its wild type relative (C2) with the exception for the lack of two SSRs (*23 and #26 in Supplementary Figure S3). Furthermore, based on the current annotations, over 85% of the total mtSSRs were located in the intergenic region, whereas this value declined to no more than 65% for the cpSSRs (Fig. 4). Nevertheless, the number of SSRs located in the coding sequence (CDS) region was similar (*P* > 0.05). A significant discrepancy in the proportion of non-CDS (mainly intron)-located cpSSRs was still highlighted (Fig. 4). Intriguingly, all of the SSRs (#25 and #26 in Fig. 3 and #26, #27 and #28 in Supplementary Figure S3) from the short single copy (SSC) region were located in the genic region. In summary, the results obtained here will also provide basic resources for the next-step applications in the evaluation of cytoplasmic genetic differences in the pepper^68^.

### Characterization of SSR motifs by different length and repeat

**Figure 5:**
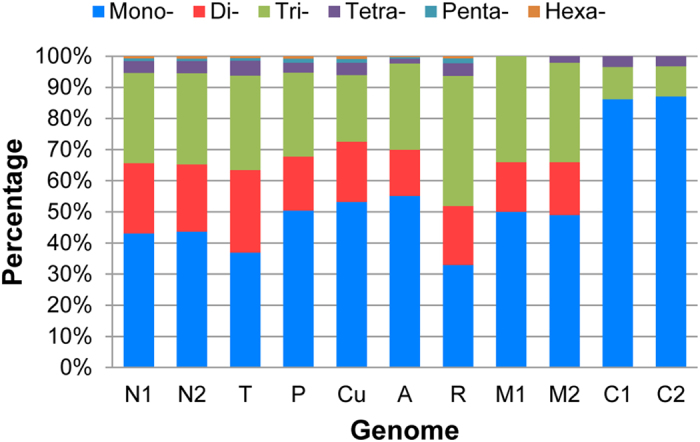
The relative proportion of SSR motifs with different lengths (1–6 bp) in eleven investigated genomes.

Data from many organisms indicated that SSR distribution across the genome is non-random^19^,^69^. Based on our search criteria, the sum of Mononucleotides (Mono-), Dinucleotides (Di-) and Trinucleotides (Tri-) accounted for the clear majority (>90%) of total SSRs for all genomes investigated in this study (Fig. 5). Of these, the Mono- was the most popular type for pepper nuclear genomes, followed by Tri-, Di-, Tetranucleotide (Tetra-), Pentanucleotide (Penta-) and Hexanucleotide (Hexa-) types. This pattern of distribution was in accordance with that found in tomato, potato, cucumber and *Arabidopsis* but not with the monocot plant (rice), in which Tri- was the main type (Fig. 5). With regard to the pepper organelle genomes, the frequency of Mono- reached higher than 80% in the pepper chloroplast whereas Tetra- was the least frequent type with percentages of 3.57% and 3.33% for C1 and C2, respectively. Furthermore, another signature of the chloroplast was the absence of Di- compared to the mitochondrial genomes. In addition, statistical results indicated an obvious trend in which the frequency of SSRs decreased with increasing repeat number regardless of species and motif length (Supplementary Table S3, Supplementary Figures S4 and S5). For example, the number of SSRs with repeat number ≤10 accounted for more than half of the total, and this rate dramatically declines to less than 4% when the repeat number is more than 20.

### Characterization of SSR motifs by classified type

**Figure 6:**
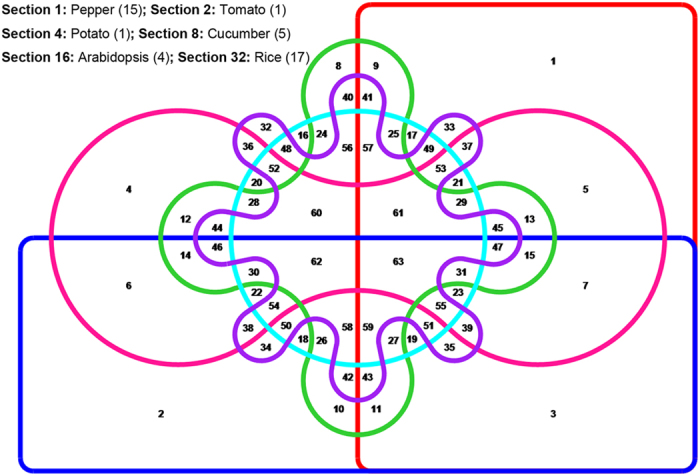
Identification of species-specific SSR motifs for the six species investigated in the present study. A set of 387 kinds of classified SSR motifs that were identified from the combination of Zunla-1(N1) and Chiltepin (N2) was used for the present analysis. Sets with red, blue, pink, green, light blue and purple colours represents pepper, tomato, potato, cucumber, *Arabidopsis* and rice, respectively. Intersection numbers are shown in the disjoint sets, and the total number of specific motifs for each species is shown with brackets in the upper left corner of the picture.

If the complementary sequence is taken into consideration, a total of 456 kinds of classified SSR motifs were detected in all genomes investigated in the present study (Table 2). The detailed frequency of classified SSR motif (1–6 bp) in different genomes is shown in the Supplementary Table S4. Out of the total of 456 kinds, 369 and 363 different motifs were identified in Zunla-1(N1) and Chiltepin (N2), respectively. Overall, we found 387 kinds of classified SSR motifs in the pepper genome. Additionally, all of the possible base combinations of Mono- (the number is 2), Di- (4) and Tri- (10) were detected in both pepper nuclear genomes as well as in all other species. However, there were inter-specific differences in both number and motif type with increasing motif length, such as Tetra-, Penta- and Hexa-. For example, 2 (CCCG/CGGG and CCGG/CCGG), 1 (CCGG/CCGG) and 6 (ACCT/AGGT, ACGC/CGTG, AGCC/CTGG, AGGC/CCTG, CCCG/CGGG and CCGG/CCGG) quadruplet motifs were not represented in potato, cucumber and *Arabidopsis*, respectively. The difference was more apparent for Penta- and Hexa-. As a result, we isolated a set of species-specific SSR motifs through integrative comparison, of which 15 motifs were found to be specific for pepper (Fig. 6, Supplementary Table S4). It is also worth mentioning that 17 species-specific SSR motifs were identified in rice (Fig. 6, Supplementary Table S4). Whether these SSR motifs play roles in the evolution of plants is unclear but we at least suggest the idea that SSR distribution and frequency are unequal^27^.

**Figure 7:**
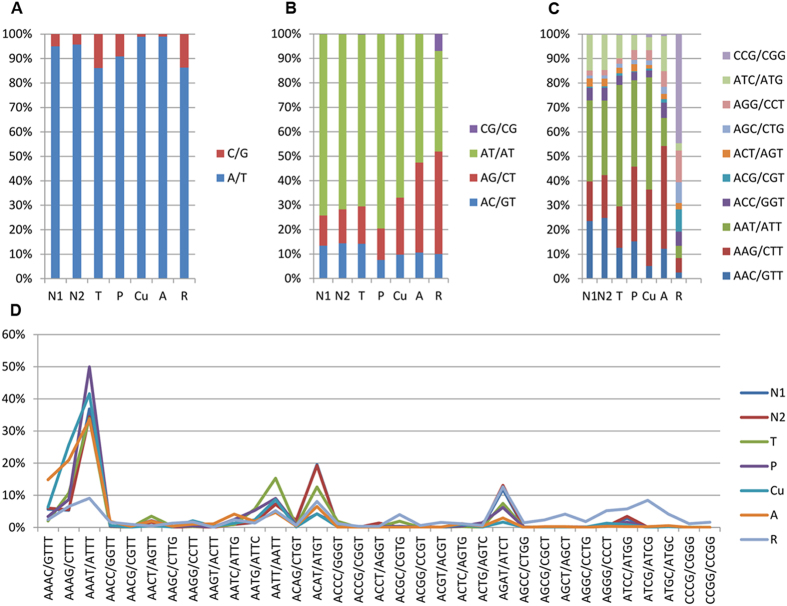
The percentage of classified SSR motifs within the corresponding type with different lengths. (**A**) Mono-; (**B**) Di-; (**C**) Tri-; (**D**) Tetra-.

In terms of the distribution of different motifs, briefly, the A/T motif not only accounted for approximately 95% of the total Mono- type in the pepper (Fig. 7A), but was also found to be the most frequent motif across all the genomes examined. In addition, the AT/AT motif, as the most predominant duplets, accounted for over 70% of the total Di- motif in pepper, tomato and potato. However, this pattern did not apply for the monocot plant rice, in which the most frequent Di- motif was AG/CT. Moreover, compared to other species such as pepper, a significantly higher percentage of CG/CG was observed in rice (Fig. 7B). Then, of the 10 different triplets, AAT/ATT was overrepresented in pepper, tomato and potato as well as in cucumber, whereas the major Tri- motif in *Arabidopsis* was AAG/CTT. Similar to the Di- motif, the GC-rich motif CCG/CGG was dramatically predominant in rice, indicating a significant difference from the dicot plant species (Fig. 7C), which was also revealed in a previous study^27^. With regard to the Tetra- motif, AAAT/ATTT was the most frequent motif in all of the genomes with the exception of rice, in which AGAT/ATCT accounted for the highest percentage (12.62%) (Fig. 7D). Additionally, AAAAT/ATTTT and AGATAT/ATATCT were the major Penta- and Hexa- motifs in pepper, respectively. The former also prevailed among Penta- motifs in tomato, potato and *Arabidopsis* but not in cucumber and rice, where AAAAG/CTTTT was overrepresented. Lastly, AACAAT/ATTGTT, AAGAGG/CCTCTT, AAAAAG/CTTTTT, AAAAAT/ATTTTT (equal in number to ACCACG/CGTGGT), and ACATAT/ATATGT predominated in the Hexa- motif of tomato, potato, cucumber, *Arabidopsis* and rice, respectively.

### Identification of unique SSR primer pairs in the Zunla-1 reference genome

In addition to understanding the characteristics of distribution, the development of molecular markers for pepper based on these SSR loci was one of the most important objectives in this study. For the purpose of convenient and efficient primer pair isolation, the compound SSRs are handled as a ‘unit’ in this section unless otherwise stated. As a result, in the Zunla-1 genome, a total of 739,723 SSR units were identified on all 13 chromosomes, including the pseudo-chromosome P0 (Table 3). The number of SSR units on each chromosome (P0-P12) ranged from 34,410 (chromosome P8) to 147,775 (chromosome P0), with an average number of 56,901.77, and the highest average density was observed on chromosome P2 (Supplementary Figure S6). Relatively, the SSR density was higher on both ends of all 12 chromosomes, which is in accordance with the distribution of protein coding genes (Fig. 1).

However, similar to the maize genome^24^,^70^, the pepper genome (Zunla-1) consists of as high as 80.90% of repetitive sequences^35^, therefore, it is better to identify *in silico* the unique SSR loci set before preparing the primer pairs for practical evaluation. First, after the size filtering standard described in the Methods section, a total of 525,563 (71.05%) SSR units, including 481,614 perfect SSRs and 43,949 compound SSRs were selected for primer pair isolation (Table 3). Of these, except for the Mono- type, the vast majority (over 98%) of perfect SSRs were kept. In contrast, more than half of the compound SSRs were filtered out because they were too long for primer design (Table 3). Then, using the Primer3 software, a total of 498,159 (94.79%) primer pairs were successfully isolated and subsequently used to align back to the Zunla-1 reference genome. Finally, a large set of 113,500 (21.60%) *in silico* unique primer pairs were obtained (Supplementary Tables S5–S9). This local database of SSR primers will undoubtedly serve as an abundant mine for molecular marker development in the pepper.

### Experimental validation of the SSR primers with a collection of pepper genotypes

To preliminarily test the usability of these SSR primers, a random set of 160 primer pairs (20 for each type) was intentionally selected from chromosome P0 to be synthesized and used for screening polymorphisms among a wide collection of 21 pepper genotypes (Table 3). These marker candidates were specially selected from chromosome P0 because they can not only to be used for possible versatile applications such as genetic diversity analysis, gene tagging and so on, but they also may be useful for anchoring some of the scaffolds that have not yet been assigned to current pepper chromosome buildings^46^. Of the initial 160 candidates, 88 (55.00%) primer pairs can specifically direct the amplification of one or two main bands (Supplementary Figure S7). In addition, out of those 88 primer pairs, 65 (73.86%) exhibited polymorphisms among the 21 pepper genotypes with the alleles per SSR marker ranging from 2 to 6 and the polymorphic information content (PIC) value for each SSR marker varying from 0.05 to 0.64 (Supplementary Table S10). Specifically, the results showed that the polymorphic rate was highest in Hexa-, followed by Penta- and C type, whereas the Tri- type exhibited the lowest polymorphic rate (Table 3). In addition, the PIC value was not significantly different between various SSR types (F = 0.59, *P* = 0.77). This information will provide a useful reference for the selection and design of further primer pairs to be tested.

**Figure 8:**
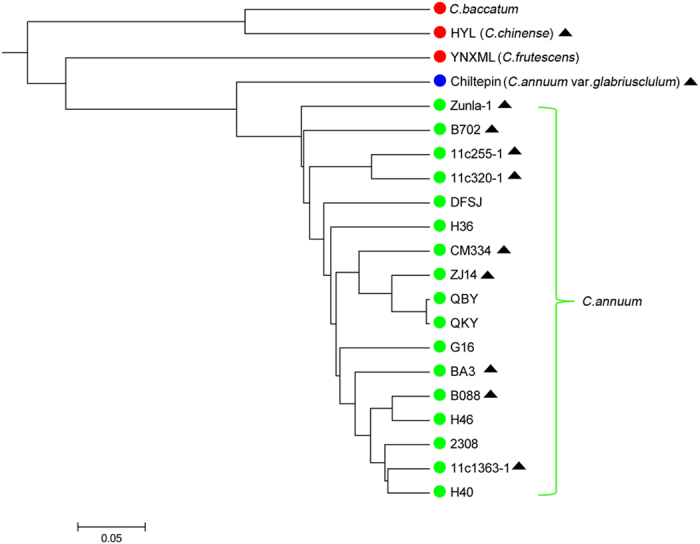
Phylogenetic tree of 21 pepper lines based on 65 polymorphic SSR markers. Genetic relationships of 11 pepper lines that were previously studied based on genome-wide SNP markers are marked with a solid black triangle suffix.

A UPGMA-phylogenetic tree (Fig. 8) was constructed based on the 65 polymorphic SSR markers. The results showed that the SSR markers developed in this study were sufficient to classify the 21 pepper lines into 3 major groups that corresponded to the domesticated species taxonomy of the *Capsicum* genus. For example, the YNXML line (*C. frutescens*) showed a closer relationship to *C. annuum* than to *C. baccatum* and *C. chinense* Jacq. This result is consistent with previous studies^71^,^72^ . In addition, the semi-wild type Chiltepin (*C. annuum var. glabriusculum*) was distinguishable from the other *C. annuum* lines. Finally, it is worth noting that the genetic relationship of 11 of the 21 pepper lines revealed by these newly developed SSR markers was comparable to our previous results inferred from the genome-wide SNPs^35^. In summary, all experimental results indicated that the SSR information obtained in this study can be useful for SSR markers development in *Capsicum*.

## Materials and Methods

### Plant materials and DNA extraction

A panel of 21 pepper genotypes (Supplementary Table S1) representing four cultivated species of the *Capsicum* genus, i.e., *C. annuum*, *C. chinense* Jacq., *C. baccatum*, *C. frutescens* and the semi-wild type, Chiltepin (*C. annuum* var*. glabriusculum*), were used to test potential application of the identified SSRs in this study. Of these, 18 genotypes were collected from a total of seven provinces/regions in China. The semi-wild type Chiltepin was collected from Queretaro in Mexico. The seeds of CM334 (Criollo de Morelos 334) and the local landrace *C. baccatum* were kindly provided by Dr. Paul W. Bosland from the Chile Pepper Institute, New Mexico State University, USA and Dr. Salvador Montes-Hernández from INIFAP-Mexico, respectively. This panel of genotypes also shows variability in fruit size, orientation, colour, pungency, and other characters. Genomic DNA was extracted from young leaves of each genotype according to the modified CTAB method^73^.

### Collection of genomic sequences from different sources

Detailed information on the sources of different genomic sequences is summarized in Supplementary Table S2. Briefly, the complete genome sequences of Zunla-1 and its wild relative Chiltepin were downloaded from the Pepper Genome Database Release 2.0 (http://peppersequence.genomics.cn/page/species/index.jsp). Recently, two complete mitochondrial genomes of pepper from the respective cytoplasmic male sterility (CMS) line ‘FS4401’ and fertile line ‘Jeju’ were published^59^. Furthermore, the complete chloroplast genome sequence of ‘FS4401’ was previously reported^61^. With the addition of another recently published chloroplast genome^60^, a total of six different genomes, including two from each nucleus, mitochondria and chloroplast, were collected for further analysis. With the aim of comparison, whole genomic sequences of an additional five plant species, including tomato (*Solanum lycopersicum*), potato (*Solanum tuberosum*), cucumber (*Cucumis sativus*), *Arabidopsis* (*Arabidopsis thaliana*) and rice (*Oryza sativa*), were also collected from the related public database (Supplementary Table S2).

### Identification and characterization of SSR loci in different genomes

The software MISA (http://pgrc.ipk-gatersleben.de/misa/) was used for SSR identification, and both perfect and compound SSRs were recorded. The detailed search criteria were as follows: (1) ten repeat units for mononucleotide (Mono-) repeats, six for dinucleotide (Di-) repeats, four for trinucleotide (Tri-), tetranucleotide (Tetra-), pentanucleotide (Penta-) and hexanucleotide (Hexa-) repeats; (2) a compound microsatellite was defined if the number of bases between two adjacent microsatellites was ≤100. The circular framework maps for four organelle genomes were drawn with the online tool, OrganellarGenomeDRAW^74^. The distribution of SSRs in genic and intergenic regions was determined based on the information of genome sequence annotations available from NCBI GenBank. The Venn diagram investigational tool VENNTURE^75^ was adopted to identify the species-specific SSR motifs.

### Identification of unique SSR primer pairs in the Zunla-1 reference genome

A homemade workflow was applied to identify the unique SSR loci in Zunla-1 genome due to the high percentage (80.90%) of repetitive sequences in the pepper genome revealed by a previous study^35^. At the same time, for the purpose of convenient and efficient primer pair isolation, the compound SSRs were handled as a ‘unit’ in the subsequent identification process unless otherwise stated. The primer modelling software Primer3 (http://www-genome.wi.mit.edu/genome_software/other/primer3.html) accompanied by Perl scripts p3_in.pl and p3_out.pl (http://pgrc.ipk-gatersleben.de/misa/primer3.html) was used for the design of primers with SSR searching results as an input.

First, because the size of some SSR units was too large to obtain the ideal size (100–500 bp) of amplification products, they were filtered by 1) Mono-unit with repeats ≤ 10; 2) other types with unit size ≤60. Then, we constructed new sequence files by expanding 500 bp based on the Zunla-1 genome on both sides of the SSR (central SSR). This process greatly decreased the reference length in primer design process. The arguments used for primer design were as follow: 1) product size: 100–500 bp; 2) primer length: 18–25 bp with optimum: 20 bp; 3) annealing temperature: 57–62 °C; 4) number of returns: 2. Second, only primers with the central SSR as a target were reserved in the process. Finally, blast was used to align the forward primer and reverse primer to the Zunla-1 reference. Unique primer pairs were defined as cases in which both forward primers and reverse primers were uniquely aligned to the genome, or in which the e-value of primary alignment was 5 times larger than that of the secondary alignment.

### Genotyping of pepper lines with a random set of selective unique SSR primers

To evaluate the utility of the above unique SSR primer pairs in *Capsicum*, a randomly selected set of 160 primer pairs was chosen to genotype the pepper lines with different sources. PCR amplification was conducted as follows: a final volume of 20 μL PCR mixture including 10 ng template DNA, 100 μM of each dNTP, 1.5 μM of each primer, 1 × reaction buffer (including Mg^2+^) and 1.0 unit of *Taq* DNA polymerase (Takara) was used for PCR reaction by an initial 3 min at 94 °C; 34 cycles of 45 s at 94 °C, 30 s at 55–58 °C, and 30 s at 72 °C, and a final 5 min at 72 °C. Then, the PCR products were electrophoresed on 6% polyacrylamide gels. A silver staining method was used to visualize bands in the gels.

### Construction of a phylogenetic tree based on polymorphic SSR markers

Based on the genotypic data, the marker analysis software PowerMarker v3.25^76^ was used to calculate the allele number and polymorphism information content (PIC) value for each polymorphic marker. Then, a phylogenetic tree was constructed based on the Nei1983’s genetic distance by the unweighted pair group method with arithmetic averages (UPGMA). Finally, the dendrogram was viewed and plotted in MEGA 6.0 software^77^.

## Additional Information

**How to cite this article**: Cheng, J. *et al.* A comprehensive characterization of simple sequence repeats in pepper genomes provides valuable resources for marker development in *Capsicum*. *Sci. Rep.* **6**, 18919; doi: 10.1038/srep18919 (2016).

## Supplementary Material

Supplementary File 1

Supplementary Table S4

Supplementary Table S5

Supplementary Table S6

Supplementary Table S7

Supplementary Table S8

Supplementary Table S9

## Figures and Tables

**Table 1:** Frequency of SSR motifs (1–6 bp) in all genomes investigated in the present study.

SSR type	Genome										
	N1	N2	M1	M2	C1	C2	Tomato	Potato	Cucumber	*Arabidopsis*	Rice
Mono-	377,376	375,358	22	23	25	27	86,922	121,911	69,085	34,751	64,831
Di-	198,033	185,688	7	8	—	—	62,486	41,875	25,117	9,375	37,370
Tri-	254,288	251,631	15	15	3	3	71,356	65,020	27,799	17,469	82,171
Tetra-	33,639	33,645	—	1	1	1	11,291	7,595	5,176	922	8,210
Penta-	6,663	6,507	—	—	—	—	1,761	3,122	1,665	351	2,958
Hexa-	6,581	6,686	—	—	—	—	1,582	2,047	1,105	178	1,393
Total number	876,580	859,515	44	47	29	31	235,398	241,570	129,947	63,046	196,933
Compound^a^	136,857	123,281	2	2	1	1	51,415	31,706	16,716	6,929	35,197
Cumulative (%)^b^	0.50	0.45	0.11	0.12	0.23	0.26	0.59	0.61	1.32	0.96	1.12
Density^c^	260.58	243.62	86.71	91.88	184.97	197.90	285.70	312.50	654.55	529.15	527.62

^a^Number of SSRs present in compound formation.

^b^The ratio of cumulative sequence length of all SSR motifs to genome size.

^c^Number of SSRs present in one million bases (SSRs/Mbp).

**Table 2:** Number of classified SSR motif types in different genomes.

Genome	Mono-	Di-	Tri-	Tetra-	Penta-	Hexa-	Total
N1	2	4	10	33	92	228	369
N2	2	4	10	33	89	225	363
Tomato	2	4	10	33	69	160	278
Potato	2	4	10	31	82	190	319
Cucumber	2	4	10	32	67	193	308
*Arabidopsis*	2	4	10	27	47	83	173
Rice	2	4	10	33	95	214	358
M1	1	3	4	0	—	—	—
M2	1	3	4	1	—	—	—
C1	1	—	2	1	—	—	—
C2	1	—	2	1	—	—	—
Total	2	4	10	33	101	306	456

“–” means not detected.

**Table 3:** Development and utility assessment of unique SSR primer pairs in the Zunla-1 genome.

		Total	Size filtering (%^b^)	Primer pairs isolation		Utility assessment			Average PIC
SSR type				Total (%^c^)	Unique (%^d^)	Selected	Specific	Polymorphic (%^e^)	
Perfect	Mono-	302,209	139,351 (46.11%)	132,118 (94.81%)	32,804 (23.54%)	20	11	7 (35.00%)	0.21
	Di-	132,938	132,802 (99.90%)	123,621 (93.09%)	26,967 (20.31%)	20	8	5 (25.00%)	0.22
	Tri-	182,359	181,411 (99.48%)	175,945 (96.99%)	38,195 (21.05%)	20	15	4 (20.00%)	0.16
	Tetra-	18,958	18,910 (99.75%)	17,935 (94.84%)	4,192 (22.17%)	20	12	8 (40.00%)	0.21
	Penta-	5,065	5,018 (99.07%)	4,839 (96.43%)	1,125 (22.42%)	20	11	11 (55.00%)	0.23
	Hexa-	4,180	4,122 (98.61%)	3,793 (92.02%)	836 (20.28%)	20	12	12 (60.00%)	0.30
C		88,729	40,559 (45.71%)	36,789 (90.70%)	8,647 (21.32%)	20	10	10 (50.00%)	0.27
C*^a^		5,285	3,390 (64.14%)	3,119 (92.01%)	734 (21.65%)	20	9	8 (40.00%)	0.30
Total		739,723	525,563 (71.05%)	498,159 (94.79%)	113,500 (21.60%)	160	88	65 (40.63%)	0.25

^a^C with an asterisk means that the number of bases interrupting two adjacent SSR motifs within a compound microsatellite is 0.

^b^The ratio of size filtering to the total number of SSR units.

^c^The ratio of successful isolation to the remainder after size filtering.

^d^The ratio of unique primer pairs to total primer pairs.

^e^The ratio of polymorphic number to number selected.
