# Supplementary material for: A comprehensive characterization of simple sequence repeats in pepper genomes provides valuable resources for marker development in Capsicum
Source: Sci Rep. 2016 Jan 7;6:18919. doi: 10.1038/srep18919 (PMC4703971; doi:10.1038/srep18919)
Supplement: Supplementary File 1 [file srep18919-s1.pdf]

A comprehensive characterization of simple sequence repeats in pepper genomes provides valuable resources for marker development in *Capsicum*

Jiaowen Cheng, Zicheng Zhao, Bo Li, Cheng Qin, Zhiming Wu, Diana L. Trejo-Saavedra, Xirong Luo, Junjie Cui, Rafael F. Rivera-Bustamante, Shuaicheng Li, Kailin Hu

SUPPLEMENTARY INFORMATION

TABLE of CONTENTS

I. Supplementary Tables 2

II. Supplementary Figures 17

Supplementary Figure S1. Overview of SSR distribution in Chiltepin genome. 17

Supplementary Figure S2. Overview of SSR distribution in pepper M2 mitochondrial genome. 18

Supplementary Figure S3. Overview of SSR distribution in pepper C2 chloroplast genome. 19

Supplementary Figure S4. Relative frequency of SSR motif with different length, by the number of repeats in six pepper genomes. 20

Supplementary Figure S5. Relative frequency of SSR motif with different length, by number of repeats in five plant species. 21

Supplementary Figure S6. Number and density comparison of SSR units on 13 chromosomes of Zunla-1 reference genome. 22

Supplementary Figure S7. Genotyping of 21 pepper lines with three newly developed polymorphic SSR markers. 23

I. Supplementary Tables

Supplementary Table S1. A representative collection of 21 pepper genotypes for availability test of SSR primer pairs.

Number	Varieties	Species	Origin of location	Phenotypic characterization of fruit
1	YNXML	*C. frutescens*	Yunnan, China	small fruit size, erect, pungent, green
2	HYL	*C. chinense*	Hainan, China	middle fruit size, pendent, pungent, yellow
3	/	*C. baccatum*	Bolivia (South America)	middle fruit size, pendent, pungent, green
4	Zunla-1	*C. annuum*	Guizhou, China	small fruit size, erect, pungent, green
5	CM334	*C. annuum*	Morelos, Mexico	small fruit size, erect, pungent, green
6	BA3	*C. annuum*	Guangdong, China	middle fruit size, pendent, pungent, green
7	B702	*C. annuum*	Guangdong, China	small fruit size, pendent, pungent, green
8	ZJ14	*C. annuum*	Guizhou, China	small fruit size, erect and fascicled, pungent, green
9	11c255-1	*C. annuum*	Beijing, China	large fruit size, pendent, non-pungent, green
10	11c320-1	*C. annuum*	Beijing, China	large fruit size, pendent, non-pungent, green
11	11c1363-1	*C. annuum*	Beijing, China	line fruit shape, pendent, pungent, green
12	QBY	*C. annuum*	Guizhou, China	small fruit size, erect, pungent, green
13	2308	*C. annuum*	Guangdong, China	middle fruit size, pendent, pungent, green
14	G16	*C. annuum*	Guangdong, China	small fruit size, erect, pungent, purple
15	QKY	*C. annuum*	Guizhou, China	small fruit size, erect, pungent, green
16	DFSJ	*C. annuum*	Guangdong, China	large fruit size, pendent, pungent, green
17	H36	*C. annuum*	Hunan, China	middle fruit size, pendent, pungent, green

18	H40	*C. annuum*	Hunan, China	middle fruit size, pendent, pungent, green
19	H46	*C. annuum*	Jiangsu, China	large fruit size, pendent, pungent, green
20	Chiltepin	*C. annuum var. glabriusculum*	Queretaro, Mexico	small fruit size, erect, pungent, green, fertile, semi-wild type
21	B088	*C. annuum*	Guangdong, China	middle fruit size, pendent, pungent, green

Supplementary Table S2. List of 11 different genomes for SSR identification in present study.

Name of variety/Species	Latin name	Genome type	Abbr.	Cromosome Number	Accession number or release version	Genome Size (bp)	References or web sources
Zunla-1	*Capsicum annuum*	Nuclear	N1	13	Release 2.0	3,363,962,270	http://peppersequence.genomics.cn/page/species/index.jsp
Chiltepin	*C. annuum var. glabriusculum*	Nuclear	N2	13	Release 2.0	3,528,040,346	http://peppersequence.genomics.cn/page/species/index.jsp
FS4401	*C. annuum*	Mitochondrial	M1	1	KJ865409	507,452	Jo et al. 2014
Jeju	*C. annuum*	Mitochondrial	M2	1	KJ865410	511,530	Jo et al. 2014
FS4401	*C. annuum*	Chloroplast	C1	1	NC_018552	156,781	Jo et al. 2011
American bird pepper	*C. annuum var. glabriusculum*	Chloroplast	C2	1	KJ619462	156,612	Zeng et al. 2014
Tomato	*Solanum lycopersicum*	Nuclear	T	13	SL2.50	823,944,053	ftp://ftp.solgenomics.net/tomato_genome/assembly/build_2.50/
Potato	*Solanum tuberosum*	Nuclear	P	13	version 4.03	773,029,444	http://solanaceae.plantbiology.msu.edu/pgsc_download.shtml
Cucumber	*Cucumis sativus*	Nuclear	Cu	8	version 2	198,529,394	ftp://www.icugi.org/pub/genome/cucumber/Chinese_long/v2/
Arabidopsis	*Arabidopsis thaliana*	Nuclear	A	5	version 10	119,146,352	ftp://ftp.arabidopsis.org/home/tai

							r/Sequences/whole_chromosomes/
Rice	*Oryza sativa*	Nuclear	R	12	IRGSP-1.0.25	373,245,530	ftp://ftp.ensemblgenomes.org/pub/plants/release-25/fasta/oryza_sativa/dna/

Supplementary Table S3. Frequency distribution of the perfect SSRs with diferent repeat number in all genomes that investigated in present study .

**Genome**	**SSR type**	**Repeat number**																	**Total**
		**4**	**5**	**6**	**7**	**8**	**9**	**10**	**11**	**12**	**13**	**14**	**15**	**16**	**17**	**18**	**19**	**20**	**>20**	
N1	Mono-	0	0	0	0	0	0	171,548	80,702	45,845	26,448	16,720	11,227	7,301	4,851	3,117	2,086	1,439	6,092	377,376
	Di-	0	0	63,109	36,006	26,573	18,510	12,943	9,325	6,885	5,417	4,387	3,504	2,698	2,159	1,758	1,466	1,047	2,246	198,033
	Tri-	156,266	44,330	20,037	10,586	5,752	3,557	2,404	1,956	1,785	1,593	936	722	604	522	471	388	319	2,060	254,288
	Tetra-	20,579	6,452	2,906	1,443	783	485	286	154	126	75	67	36	34	28	15	17	16	137	33,639
	Penta-	4,989	1,011	282	125	82	34	30	20	15	12	18	6	1	7	5	4	0	22	6,663
	Hexa-	4,424	1,117	434	263	103	87	44	23	24	12	10	4	5	6	3	2	0	20	6,581
	**Total**																			**876,580**
N2	Mono-	0	0	0	0	0	0	177,504	81,962	44,890	25,252	15,244	9,925	6,376	3,895	2,559	1,716	1,182	4,853	375,358
	Di-	0	0	63,481	34,986	26,022	18,508	12,693	8,476	5,849	4,182	2,824	2,095	1,584	1,168	902	740	558	1,620	185,688
	Tri-	158,105	43,724	19,863	10,203	5,552	3,486	2,265	1,600	1,281	1,043	895	538	380	336	258	223	182	1,697	251,631
	Tetra-	20,563	6,530	2,932	1,428	735	496	307	160	98	74	33	33	26	28	12	11	18	161	33,645
	Penta-	4,983	982	258	92	59	40	19	11	7	8	4	8	3	3	1	4	2	23	6,507
	Hexa-	4,581	1,145	426	247	88	49	35	27	16	10	4	8	8	1	4	2	1	34	6,686
	**Total**																			**859,515**
M1	Mono-	0	0	0	0	0	0	13	7	1	0	0	0	1	0	0	0	0	0	22
	Di-	0	0	6	1	0	0	0	0	0	0	0	0	0	0	0	0	0	0	7

	Tri-	12	3	0	0	0	0	0	0	0	0	0	0	0	0	0	0	0	0	15
	Tetra-	-	-	-	-	-	-	-	-	-	-	-	-	-	-	-	-	-	-	-
	Penta-	-	-	-	-	-	-	-	-	-	-	-	-	-	-	-	-	-	-	-
	Hexa-	-	-	-	-	-	-	-	-	-	-	-	-	-	-	-	-	-	-	-
	**Total**																			**44**
M2	Mono-	0	0	0	0	0	0	13	5	4	0	0	0	1	0	0	0	0	0	23
	Di-	0	0	7	1	0	0	0	0	0	0	0	0	0	0	0	0	0	0	8
	Tri-	12	3	0	0	0	0	0	0	0	0	0	0	0	0	0	0	0	0	15
	Tetra-	1	0	0	0	0	0	0	0	0	0	0	0	0	0	0	0	0	0	1
	Penta-	-	-	-	-	-	-	-	-	-	-	-	-	-	-	-	-	-	-	-
	Hexa-	-	-	-	-	-	-	-	-	-	-	-	-	-	-	-	-	-	-	-
	**Total**																			**47**
C1	Mono-	0	0	0	0	0	0	12	5	2	2	1	1	0	2	0	0	0	0	25
	Di-	-	-	-	-	-	-	-	-	-	-	-	-	-	-	-	-	-	-	-
	Tri-	2	1	0	0	0	0	0	0	0	0	0	0	0	0	0	0	0	0	3
	Tetra-	1	0	0	0	0	0	0	0	0	0	0	0	0	0	0	0	0	0	1
	Penta-	-	-	-	-	-	-	-	-	-	-	-	-	-	-	-	-	-	-	-
	Hexa-	-	-	-	-	-	-	-	-	-	-	-	-	-	-	-	-	-	-	-
	**Total**																			**29**
C2	Mono-	0	0	0	0	0	0	12	7	2	0	1	1	0	0	0	3	1	0	27
	Di-	-	-	-	-	-	-	-	-	-	-	-	-	-	-	-	-	-	-	-
	Tri-	2	1	0	0	0	0	0	0	0	0	0	0	0	0	0	0	0	0	3
	Tetra-	1	0	0	0	0	0	0	0	0	0	0	0	0	0	0	0	0	0	1
	Penta-	-	-	-	-	-	-	-	-	-	-	-	-	-	-	-	-	-	-	-
	Hexa-	-	-	-	-	-	-	-	-	-	-	-	-	-	-	-	-	-	-	-
	**Total**																			**31**

T	Mono-	0	0	0	0	0	0	44,244	21,380	8,573	6,025	2,456	1,814	917	609	317	209	138	240	86,922
	Di-	0	0	14,890	8,662	6,477	4,954	3,723	2,711	2,307	1,974	1,833	1,553	1,417	1,317	1,169	1,113	1,054	7,332	62,486
	Tri-	40,456	12,890	6,568	3,621	2,245	1,604	1,097	757	542	414	275	194	183	121	98	70	59	162	71,356
	Tetra-	8,025	1,881	616	325	137	100	73	43	34	14	12	4	6	1	3	1	4	12	11,291
	Penta-	1,461	192	52	15	15	6	5	3	3	2	1	4	0	0	1	0	0	1	1,761
	Hexa-	1,145	242	107	39	15	18	6	5	1	0	0	1	0	0	2	0	0	1	1,582
	**Total**																			**235,398**
P	Mono-	0	0	0	0	0	0	59,108	24,070	13,117	7,655	5,096	3,613	2,428	1,731	1,255	889	643	2,306	121,911
	Di-	0	0	12,754	7,303	5,125	3,825	3,025	2,302	1,902	1,502	1,155	1,087	633	316	217	129	98	502	41,875
	Tri-	41,162	12,160	5,188	2,511	1,459	796	488	275	107	54	50	18	18	18	13	9	14	680	65,020
	Tetra-	5,876	1,185	314	117	32	13	7	4	2	2	3	2	2	3	0	0	0	33	7,595
	Penta-	2,638	367	75	21	3	2	1	0	0	1	2	0	0	0	1	1	0	10	3,122
	Hexa-	1,483	318	93	17	10	4	3	7	9	3	1	2	3	4	2	3	1	84	2,047
	**Total**																			**241,570**
Cu	Mono-	0	0	0	0	0	0	26,424	13,286	7,788	5,195	3,738	2,925	2,569	2,084	1,451	969	616	2,040	69,085
	Di-	0	0	8,869	5,552	4,223	2,718	1,526	755	394	229	153	74	62	32	25	5	6	494	25,117
	Tri-	17,165	5,486	2,555	1,269	624	204	99	43	20	16	7	18	8	5	4	4	3	269	27,799
	Tetra-	3,787	934	262	71	23	9	8	5	3	2	4	1	0	1	2	2	2	60	5,176
	Penta-	1,273	286	38	4	6	4	0	7	2	2	0	2	1	1	1	0	2	36	1,665
	Hexa-	854	155	11	3	6	1	5	1	1	1	2	3	0	0	0	0	0	62	1,105
	**Total**																			**129,947**
A	Mono-	0	0	0	0	0	0	14,155	6,946	3,839	2,441	1,735	1,335	1,087	783	589	441	358	1,042	34,751
	Di-	0	0	3,121	1,660	1,213	845	551	381	284	196	199	157	139	98	80	64	58	329	9,375
	Tri-	11,885	3,238	1,270	515	217	95	69	50	29	14	12	17	16	6	5	0	4	27	17,469
	Tetra-	753	119	35	7	2	1	1	0	0	1	0	0	0	0	0	0	2	1	922
	Penta-	310	29	7	3	2	0	0	0	0	0	0	0	0	0	0	0	0	0	351

	Hexa-	121	38	9	2	3	1	2	0	0	0	1	0	0	1	0	0	0	0	178
	**Total**																			**63,046**
R	Mono-	0	0	0	0	0	0	36,537	11,850	6,236	3,467	2,176	1,416	883	598	486	332	243	607	64,831
	Di-	0	0	12,817	6,790	3,995	2,626	1,743	1,154	845	659	553	516	424	370	330	328	294	3,926	37,370
	Tri-	52,359	17,919	6,730	2,797	987	437	177	107	71	52	35	44	29	31	36	32	27	301	82,171
	Tetra-	5,641	1,474	447	195	111	73	59	41	27	25	24	18	9	9	11	4	5	37	8,210
	Penta-	2,353	484	95	18	3	0	0	3	0	0	0	0	0	0	1	0	0	1	2,958
	Hexa-	1,133	182	47	14	6	1	1	2	2	2	0	1	0	1	0	0	0	1	1,393
	**Total**																			**196,933**

—: this type of SSR were not detected in the present study.

Supplementary Table S4. Frequency of classified SSR motif (1~6bp) in different genomes[a].

This table is provided in the form of a single file.

Supplementary Table S5. List of 11,772 unique SSR primer pairs identified from the chromosome P0 of Zunla-1 reference genome.

This table is provided in the form of a single file.

Supplementary Table S6. List of 31,111 unique SSR primer pairs identified from the chromosomes P1 to P3 of Zunla-1 reference genome.

This table is provided in the form of a single file.

Supplementary Table S7. List of 24,982 unique SSR primer pairs identified from the chromosomes P4 to P6 of Zunla-1 reference genome.

This table is provided in the form of a single file.

Supplementary Table S8. List of 22,024 unique SSR primer pairs identified from the chromosomes P7 to P9 of Zunla-1 reference genome.

This table is provided in the form of a single file.

Supplementary Table S9. List of 23611 unique SSR primer pairs identified from the chromosomes P10 to P12 of Zunla-1 reference genome.

This table is provided in the form of a single file.

Supplementary Table S10.Information of the 88 newly developed SSR markers from chromosome P0 in present study.

Nomenclature[a]	SSR type	SSR	size	Chr	start	end	Forward primer (5'-3')	Tm(℃)	Reverse primer (5'-3')	Tm(℃)
SSR_P0_163110979[#]	p6	(TTTAAT)4	24	P0	163,110,979	163,111,002	ACGGTTGGTGGACTCTCATT	58.94	GGGATTTGCGAAAACTTGCA	57.85
SSR_P0_164189256[#]	p5	(CATGT)4	20	P0	164,189,256	164,189,275	CGCACCTTTTCCGACTCTTT	58.77	AGAAAGTCACTCCTCTCCGC	59.11
SSR_P0_438581243[#]	p6	(TATTTT)4	24	P0	438,581,243	438,581,266	AGTCGACTTACAGCTGAGGT	58.09	ACCATATAATCACGCCTCAAGA	57.04
SSR_P0_449855951[#]	p6	(CCATCT)9	54	P0	449,855,951	449,856,004	GCCGTCACCTTCGATTACAC	59.00	TGTAATCGACGGTGCTAGCA	59.19
SSR_P0_456808942[#]	p6	(CAACTT)5	30	P0	456,808,942	456,808,971	AAGCTCAGGACTCGTTTCA	57.59	ACGTGAATGAGCCAAGT	57.67

							TT		ATGT	
SSR_P0_462067694[#]	p6	(ATATTC)4	24	P0	462,067,694	462,067,717	TACATCCGCCTCTGAACTCC	58.89	TTTACTTGTGGTTTCGGAAGC	57.03
SSR_P0_471765445[#]	c*	(TA)6(ATAA)5*	29	P0	471,765,445	471,765,473	TGGCTGTTACCGGTCATCTT	59.02	TGCGTAACAGAGGATTGCAG	58.27
SSR_P0_480209261[#]	c*	(AT)6(T)13*	24	P0	480,209,261	480,209,284	ACAAGTATGGAGGGAGCAAATT	57.68	CTCCCGAGGCCCCATATATC	58.88
SSR_P0_480718061[#]	p4	(ATAA)4	16	P0	480,718,061	480,718,076	AGGGGTGTGACATCGTTCAT	59.02	CCAGAATCACTACCCAAGGC	57.96
SSR_P0_480905753[#]	c*	(G)16(GAG)4*	27	P0	480,905,753	480,905,779	AACTGTGCTCCTCCCTTCTC	59.02	TCGCCATTCACTTCACTCCT	59.02
SSR_P0_480940810	p4	(TATT)4	16	P0	480,940,810	480,940,825	TCAAGACTTTTCATCACACCAGA	57.97	AGGAATTGAAGGATCGGAGGG	59.23
SSR_P0_487884506[#]	p4	(TAAT)4	16	P0	487,884,506	487,884,521	GCCCGTCACTAAAAGTCTCC	58.28	TCTGGAATGGCTGTACTACCA	58.17
SSR_P0_488531631[#]	p2	(TA)7	14	P0	488,531,631	488,531,644	CCTAGAATGACCCCGACTGT	58.51	GGGGCTCCATACCAGAAAGA	58.79
SSR_P0_489955492	p2	(AT)6	12	P0	489,955,492	489,955,503	CATCACACACCCCAATGCC	59.11	TGATGATACATATGTCTGCCAAGA	57.58
SSR_P0_491728482[#]	p2	(AG)6	12	P0	491,728,482	491,728,493	ACTAACTGAAACGGCTGACAC	58.52	TCGGTGTCCAATGGTAAGCT	59.02
SSR_P0_491740645[#]	p6	(TGCTGA)4	24	P0	491,740,645	491,740,668	CGGTGGCTAGAGAGGAAGAG	58.97	AATCCGACTCACCTTCAGCA	59.02
SSR_P0_493610495[#]	c	(AAT)5tataataac(AAT)6	42	P0	493,610,495	493,610,536	TGTGGGATTGGCGCTTTAAG	58.83	TGTATACAAAAGCGTGGCGG	58.92

SSR_P0_494287518	p1	(T)10	10	P0	494,287,518	494,287,527	TGTGGTTTGGCTTTGAAGGG	58.88	AGTCACTTAACCATTTTGACGTGA	58.88
SSR_P0_494755411#	c	(A)13gcacaacaaac(AAT)6	42	P0	494,755,411	494,755,452	GTGAAGTCCGGAGAGAGTGA	58.46	GGCCGAGGATATGAAGGTGA	58.95
SSR_P0_494756680#	c	(ATTT)4cctc(T)12	32	P0	494,756,680	494,756,711	ACGCGCTTTGTCATGTGTAA	58.78	ACGTGTTAGCCTACGGTGAA	59.04
SSR_P0_494816190#	p1	(T)10	10	P0	494,816,190	494,816,199	AGCTTTTAAGGATGGTGGCA	57.40	TGAGATCCAATGAGAATAAAATGGG	57.17
SSR_P0_495003120#	p3	(GAA)4	12	P0	495,003,120	495,003,131	GTTGGGTTGGCAATGGACAT	59.02	CTCGACTTGTCGCTTAGCAC	59.01
SSR_P0_495031739	p3	(AAC)4	12	P0	495,031,739	495,031,750	GCGACGCCTTTCAGATTTCA	59.20	CTTCCTAGACCCCACCTGTG	59.10
SSR_P0_495114418#	c	(TA)6cac(AT)7	29	P0	495,114,418	495,114,446	GGTTGGGCTTGATGACTGTG	59.12	TCTTATCTCTCCGCACCGAC	58.98
SSR_P0_496173676#	p1	(T)10	10	P0	496,173,676	496,173,685	TCCATACCGCGACAACAAAC	58.85	GCTCGTGTTGTTTGCGTTTT	58.74
SSR_P0_496193829#	p3	(TTC)4	12	P0	496,193,829	496,193,840	TGTCCTCCTCCTTCTCTTGT	57.30	GCAGCGACAGGAGTTGAAAA	59.06
SSR_P0_496530536	p3	(ATC)4	12	P0	496,530,536	496,530,547	TTTTCATGGCCAGGACTTCG	58.47	ATTTGCGTCTGTTCGGTGTT	58.70
SSR_P0_496647902#	p3	(AAT)4	12	P0	496,647,902	496,647,913	TGTCGCTGTGCATGTAGTTG	58.85	CCGACATTGGAAACCGTGTT	59.05
SSR_P0_498360802#	p4	(AAAT)4	16	P0	498,360,802	498,360,817	GCCATGCAAACAGGAAAGGA	59.03	TTGGGGTGGGTATGAAAGGT	58.54
SSR_P0_503923900#	p6	(AAAAGG)	30	P0	503,923,900	503,923,929	TTGGGATCTTCGTCAGCAG	59.02	TGTCATGGGGTAGACAG	57.08

		5					T		AGA	
SSR_P0_506473556[#]	c*	(CTAT)8(TAT)7*	50	P0	506,473,556	506,473,605	TAATCACTCCACAGGCTGCA	59.02	CCACACACGTTTTAGGGAAGA	58.16
SSR_P0_512937722[#]	p6	(TTTGAA)4	24	P0	512,937,722	512,937,745	TGTTGCTCGATTAGGCTGGA	59.10	GTTTTCAAGCAGTGCCTCGA	59.06
SSR_P0_526557141[#]	p5	(TTTTG)4	20	P0	526,557,141	526,557,160	TTCGGCTGGAAAAGGATTGG	58.46	CGAGGGAAGTAGGGTTAGGG	58.59
SSR_P0_530599646[#]	p5	(TGTTC)5	25	P0	530,599,646	530,599,670	TTAGGGGCCCAACAGAAGAG	59.01	CTCATTCGTGTCGGTGTTGG	59.21
SSR_P0_538786861[#]	p6	(TTCAGT)4	24	P0	538,786,861	538,786,884	CACTGCTCAAACACCACGAA	58.99	ATAGTTCCAGCGCGCAATAC	58.78
SSR_P0_547445673[#]	p5	(TAAAA)4	20	P0	547,445,673	547,445,692	TTCCACCCTTACAGCTGAGG	59.02	AAGAAAGGGGTGGGGTATGG	58.99
SSR_P0_570807688[#]	c*	(TAGA)5(AG)8*	33	P0	570,807,688	570,807,720	GTAGGGCAAGGAGAGAGCAA	59.10	GATCCCAACTGAGCTCCTGT	59.09
SSR_P0_573751529[#]	p5	(TTTGT)4	20	P0	573,751,529	573,751,548	CCTAAGTCAATCCGGCTCCT	58.88	CTTGGGCTCAACGTATGGTG	58.91
SSR_P0_580381951[#]	p4	(TTTA)6	24	P0	580,381,951	580,381,974	TCAAATGGCTTCTGTTGAGGT	57.77	AGAGTATCTGACACGCACCA	58.45
SSR_P0_587527153[#]	p6	(AATAAA)4	24	P0	587,527,153	587,527,176	TCCAAACTACAAGCCTGCCT	59.23	TGACACCAAGCGACAACTTT	58.26
SSR_P0_587565701[#]	p4	(AATA)4	16	P0	587,565,701	587,565,716	GCCGGTGATTTAATATTTTGTGCA	58.89	ACAACTAAGCTGGTGGGACA	58.86
SSR_P0_593258174[#]	p4	(AACC)4	16	P0	593,258,174	593,258,189	CCATGCCCTAAACGTGACAG	58.91	CTTAGCCATAGTCGGGAGCA	58.96

SSR_P0_595611863[#]	c	(TA)7ttg(AT)7	31	P0	595,611,863	595,611,893	GCGGCATACAAAGGAAGGAG	58.98	GAGTAGACCCTGGGCAATGA	58.80
SSR_P0_596062534[#]	c	(AT)12(ACAT)4	40	P0	596,062,534	596,062,573	TCAACAGTACGGCAGGACAT	59.03	ATTCTGAACGCGTGCTTCTC	58.93
SSR_P0_597136478[#]	p1	(A)10	10	P0	597,136,478	597,136,487	GGCAATTGTCTAGGGCTTCA	57.87	TCGTGCATCTCAATCATATCAGT	57.79
SSR_P0_597243550[#]	p1	(A)10	10	P0	597,243,550	597,243,559	TGATGTGCAGTGGATGGGTA	58.72	AGACTGAGGGACGGGTAGTA	58.71
SSR_P0_597479614[#]	p1	(A)10	10	P0	597,479,614	597,479,623	ACACAACCAACCCCATAAAGTG	59.04	GCGTGAGGAGAGGAGAATGA	58.89
SSR_P0_597975613	p1	(T)10	10	P0	597,975,613	597,975,622	AATTCCCCTGCCTTGTTTGC	59.31	AGAGGATGATGGTGTTGGCT	58.71
SSR_P0_599973541	p4	(GAAA)4	16	P0	599,973,541	599,973,556	CCAACAATCACTGCAGGCTT	59.04	GTGCCAATTACGTCCGGTTC	59.56
SSR_P0_601491402	p2	(TA)7	14	P0	601,491,402	601,491,415	AGTTCGACCCTTGTGTCACA	59.17	AGAGGTGGAAGGTTGGAGTC	58.65
SSR_P0_603004334	p2	(AT)7	14	P0	603,004,334	603,004,347	AATGATTGGACGTGGCATGC	59.54	TCTGGTGGCAGTGGACTATG	59.10
SSR_P0_604165084[#]	p2	(AT)7	14	P0	604,165,084	604,165,097	CGCCGGACTTAACCTGAAAC	59.21	GGAATTCACTGGGGCAATGG	59.17
SSR_P0_606387537	p3	(TTA)6	18	P0	606,387,537	606,387,554	TTGTTCCCTCAAGTGGACCA	58.78	CGTCGGAGTGGCTAAAATGA	58.00
SSR_P0_606517693	p3	(TGT)5	15	P0	606,517,693	606,517,707	TGAGGGTCTTTCGGGAAACA	58.87	GACTGCATTTTCCCACTAACCA	58.85
SSR_P0_606531330	p3	(TGT)4	12	P0	606,531,330	606,531,341	ACAATCTCTCTGCCCCACAA	58.93	GAGCACCCTTTACTCCTCGT	59.10

SSR_P0_607395188	p3	(TTG)4	12	P0	607,395,188	607,395,199	TGGTTTCTCTCAACTCTGCCA	59.23	GGCGAATCGGATGTCAACAA	58.92
SSR_P0_608011956	p3	(CAA)5	15	P0	608,011,956	608,011,970	TTAGCGTGACATCTTTGCCG	58.92	ACGCCTGGAATTACACTGGT	59.31
SSR_P0_609133294#	c*	(A)10(AT)7*	23	P0	609,133,294	609,133,316	ACGGGATGATATTATTCGAATGGA	57.71	TATCAACTGGGCCGGATCTC	58.95
SSR_P0_610565641#	c	(AT)7c(TA)7	29	P0	610,565,641	610,565,669	TCCGGCAAGTACTTACCCTT	58.35	CCGCCCCAAACTAGACAGAT	59.46
SSR_P0_635258944#	p5	(TAGTG)4	20	P0	635,258,944	635,258,963	CACCCGCAACCAAGATCAAA	59.04	GCCACCATGTAAGCTTCCAC	59.19
SSR_P0_643195941#	c*	(A)10(AAT)5*	23	P0	643,195,941	643,195,963	TGAGCCTCATATTGTGCCCT	58.78	AGGTGCCACGTCCAATAGAT	58.80
SSR_P0_647206762#	p5	(AAGAG)4	20	P0	647,206,762	647,206,781	TTTCCCCGGCAAAATATCCA	57.47	CCCCATCTTGCAGGAATTCG	58.97
SSR_P0_655489419#	p5	(TAATT)6	30	P0	655,489,419	655,489,448	TCTACGACTACAGCGTACCT	57.32	CGACCTCAACAAGACTCTATGC	58.82
SSR_P0_672904565	c*	(A)10(AAAAG)4*	26	P0	672,904,565	672,904,590	CGCAGAAATACGTTGGGCTA	58.35	GCCGACGATGATCTACTCCT	58.76
SSR_P0_684352014#	p5	(AAAAC)4	20	P0	684,352,014	684,352,033	CACGAGCTCAAGGTGAATCA	57.92	TGGACCTCTTGCATCCGTAA	58.73
SSR_P0_705767169#	p5	(TTTAT)4	20	P0	705,767,169	705,767,188	TGTCCACCATTTTCCTGTTGT	57.98	AAGCAAGCAAGAGAGTCAGC	58.48
SSR_P0_706518612#	c*	(A)15(AT)8*	30	P0	706,518,612	706,518,641	GCTAGGGCCACGACTAAGAT	58.96	AGGCATACTTAAAGCACGCT	57.60
SSR_P0_706679929#	p6	(ATCTGA)4	24	P0	706,679,929	706,679,952	GGGGCAGAATTGTACAAGCC	59.18	ACGTCATCCATCAGACCACA	58.73

SSR_P0_707670527#	p5	(GAAAA)4	20	P0	707,670,527	707,670,546	CACTTGACTCACAGCGATGG	58.93	CCACCCGCTGATAACCAAGA	59.75
SSR_P0_712009853#	c	(ATT)5t(ATA)4	28	P0	712,009,853	712,009,880	ATGCTTCGTCCTCTGTCCTC	59.18	AGAGGTCAACTAGCCAAAGAAA	57.29
SSR_P0_712433624#	c	(CAC)4aaattttagtagattcttaatcattggtactac(GTT)4	57	P0	712,433,624	712,433,680	CCCCTCCCTCAATTCACCTT	59.00	ATGAACAAGAATCCAAACAAGCT	57.20
SSR_P0_712988435#	p6	(AAAAAT)4	24	P0	712,988,435	712,988,458	TGCTCCGACACATATGGAGT	58.52	GACGCAACACCAGACAACAT	59.06
SSR_P0_713201073#	c	(ATAC)6atatatat(AC)7	46	P0	713,201,073	713,201,118	ATCCTTCATCCCACGTCGAA	58.81	ATTTCCCACACCTACTCGCA	59.02
SSR_P0_713466835	p4	(AAAT)4	16	P0	713,466,835	713,466,850	TCTGCTTCAACACTATTCAGACA	57.79	ACTGCTCCTCTCCGAAGATG	58.89
SSR_P0_713643490	p4	(TAAA)4	16	P0	713,643,490	713,643,505	TGAAGTAGACAAATCACCGACT	57.13	AAGACATGGGCGGAGGAAAA	59.60
SSR_P0_714045050#	p4	(AAAT)5	20	P0	714,045,050	714,045,069	TAATGCCTCCCTGATACCCC	58.26	TGCTACCATCTTGCGTGATC	58.06
SSR_P0_714546534#	p2	(TA)7	14	P0	714,546,534	714,546,547	ATGAGAAGGGGTGTACGAGC	59.18	GAAAAGGTTGGTGTTCGGCT	58.97
SSR_P0_714547079#	p3	(CAC)4	12	P0	714,547,079	714,547,090	GCCGAACACCAACCTTTTCT	58.97	AGTTGAAGGGTCTGGCTTGA	58.86
SSR_P0_714549623#	p2	(AT)6	12	P0	714,549,623	714,549,634	AGAAGGATGAATCGGCCCTT	58.78	ATCGTCATCATGGCCTAGGG	59.02
SSR_P0_714550164	p3	(TGA)4	12	P0	714,550,164	714,550,175	CGTAACGGAATCCTACCACC	57.79	TTCTCCGACATGACTGCTCA	58.74

SSR_P0_714575011	p1	(A)10	10	P0	714,575,011	714,575,020	GTTCAGGGTGAGGAGTGAGG	59.39	TGTTTTGTGGTCAGGGTGTG	58.53
SSR_P0_714623683#	p4	(ATGT)10	40	P0	714,623,683	714,623,722	TGTCAACAAGGCATGACTTTGA	58.71	ACTAGCGTGTACCAGTTGCT	59.03
SSR_P0_714657262	p1	(A)10	10	P0	714,657,262	714,657,271	GTATCGTGCCAGCAAGTACA	58.00	ATTGGTGGTGGTCTGGGTAG	59.01
SSR_P0_714673559	p3	(CAA)4	12	P0	714,673,559	714,673,570	TTGGCATGTGTCCTATCGGT	59.09	AGCTTCGGAATTAGGGGTCT	58.12
SSR_P0_714675881	p3	(TGC)4	12	P0	714,675,881	714,675,892	CCTGTATCTTCCTCGTCCCC	58.96	TCCACCGGAGACAGACTTTC	59.03
SSR_P0_714679449#	p1	(T)10	10	P0	714,679,449	714,679,458	CTGGTTCCCTCTTCCTCTCC	58.80	CCAGGAAGGCTCTTAAGGGT	58.71
SSR_P0_714680259	p3	(TCA)4	12	P0	714,680,259	714,680,270	AGGCTCTCACGCTTCTCTAC	58.90	TGCATTGAAGGAGGCTTTGT	58.00
SSR_P0_714733497#	p1	(T)10	10	P0	714,733,497	714,733,506	ACGCAAATTTCACACCTAAGAGA	58.61	TGGGAGAGACTTAAGGAGAGG	57.61

[a]The 65 polymorphic SSR markers were labeled with #.

II. Supplementary Figures

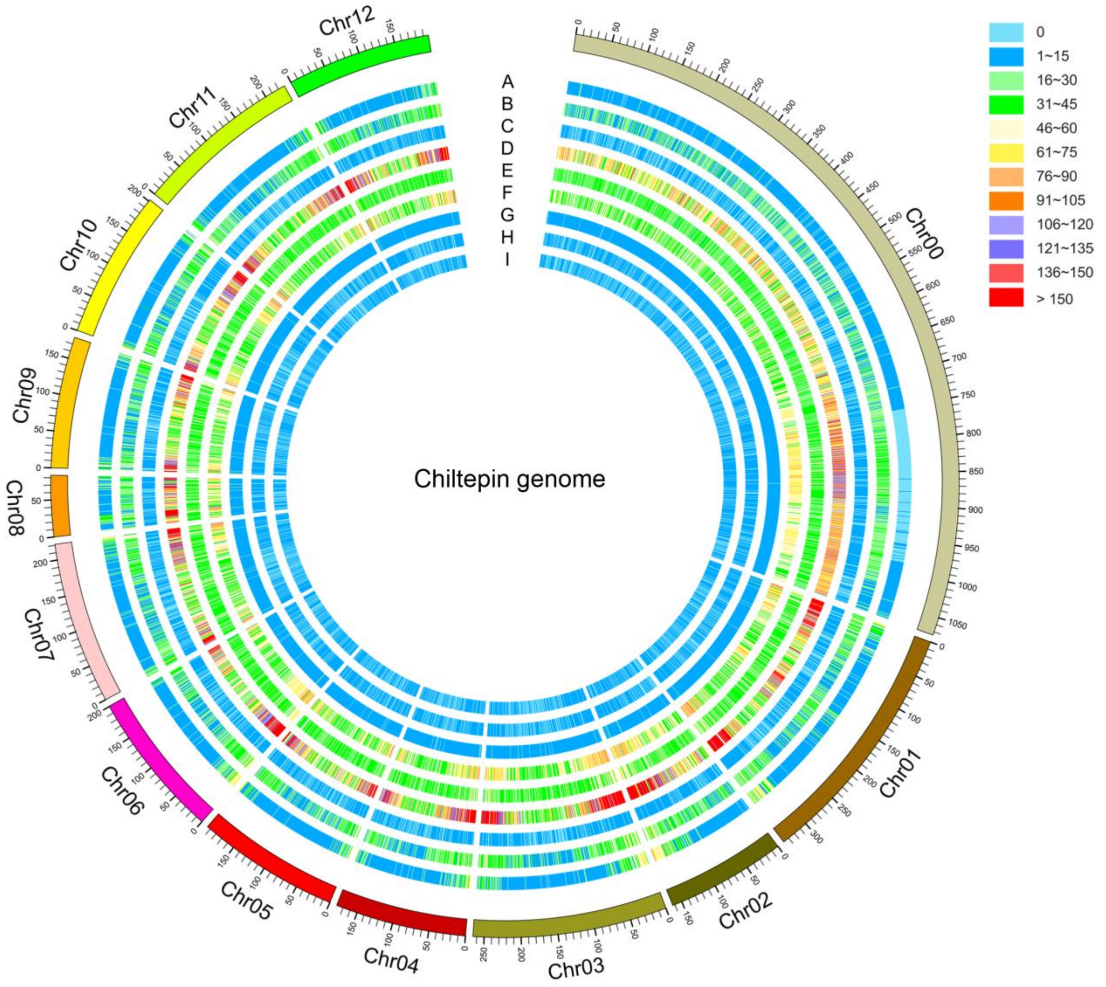

Supplementary Figure S1. Overview of SSR distribution in Chiltepin genome.

A total of 859,515 SSR loci with 123,281 presented in compound formation (C and C*), which form into 736,234 SSR units, were identified in the Chiltepin genome. The various number of SSR units and protein coding genes in each window size of 1000kb were used for drawing this picture and showed with different colours. Track A shows the gene density, tracks B to I refer to the C, C*, Mono-, Di-, Tri-, Tetra-, Penta-, Hexa- type, respectively.

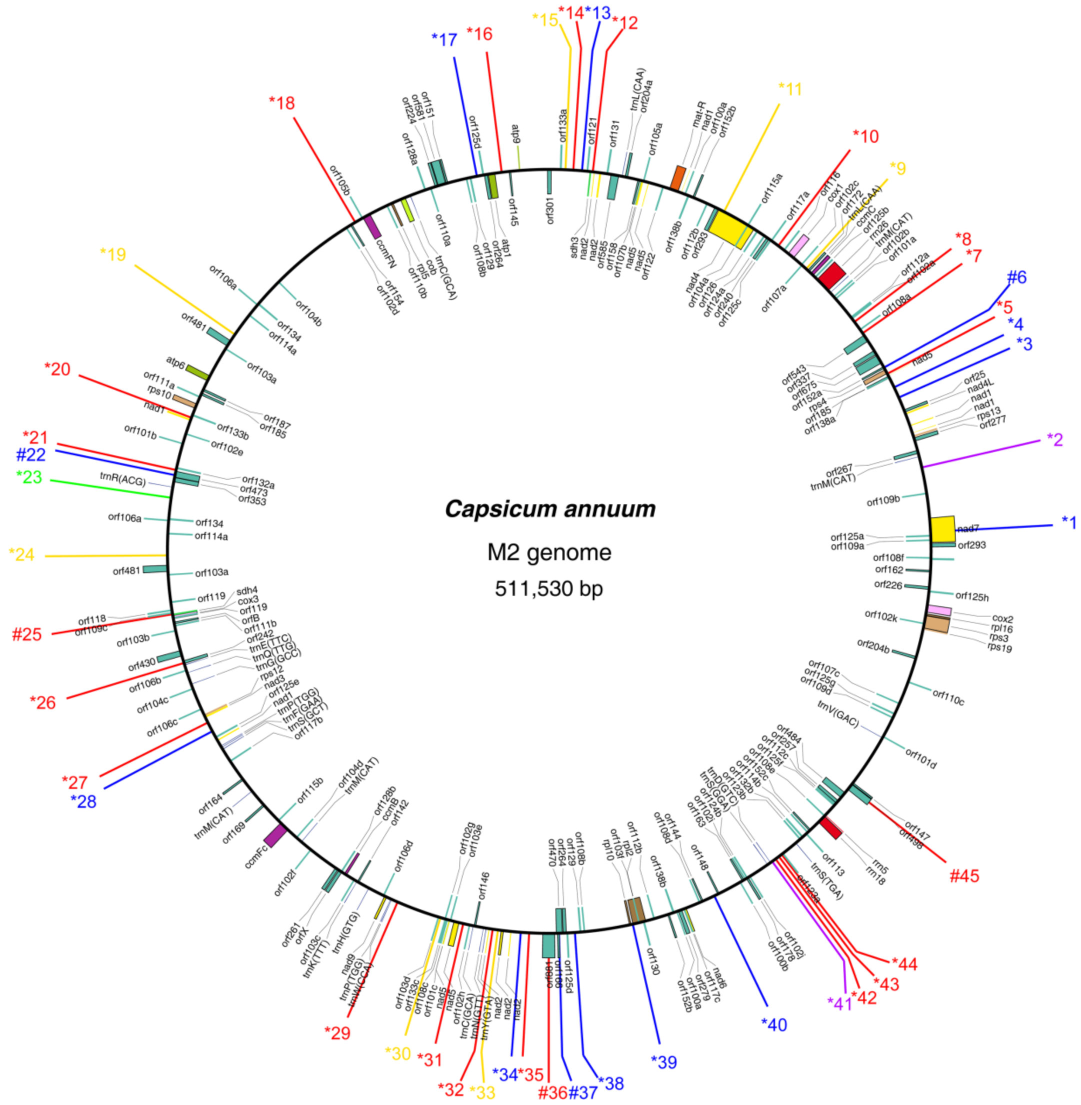

Supplementary Figure S2. Overview of SSR distribution in pepper M2 mitochondrial genome.

The M2 genome refers to the mitochondrial genome of pepper line ‘Jeju’ (*Capsicum annuum*). Perfect SSRs with 1, 2, 3 and 4 bp length of motif were represented by red, yellow, blue and green lines, respectively. And compound SSRs were showed with purple lines. Number with * and # in the front means that the corresponding SSRs were located in the intergenic and genic region, respectively.

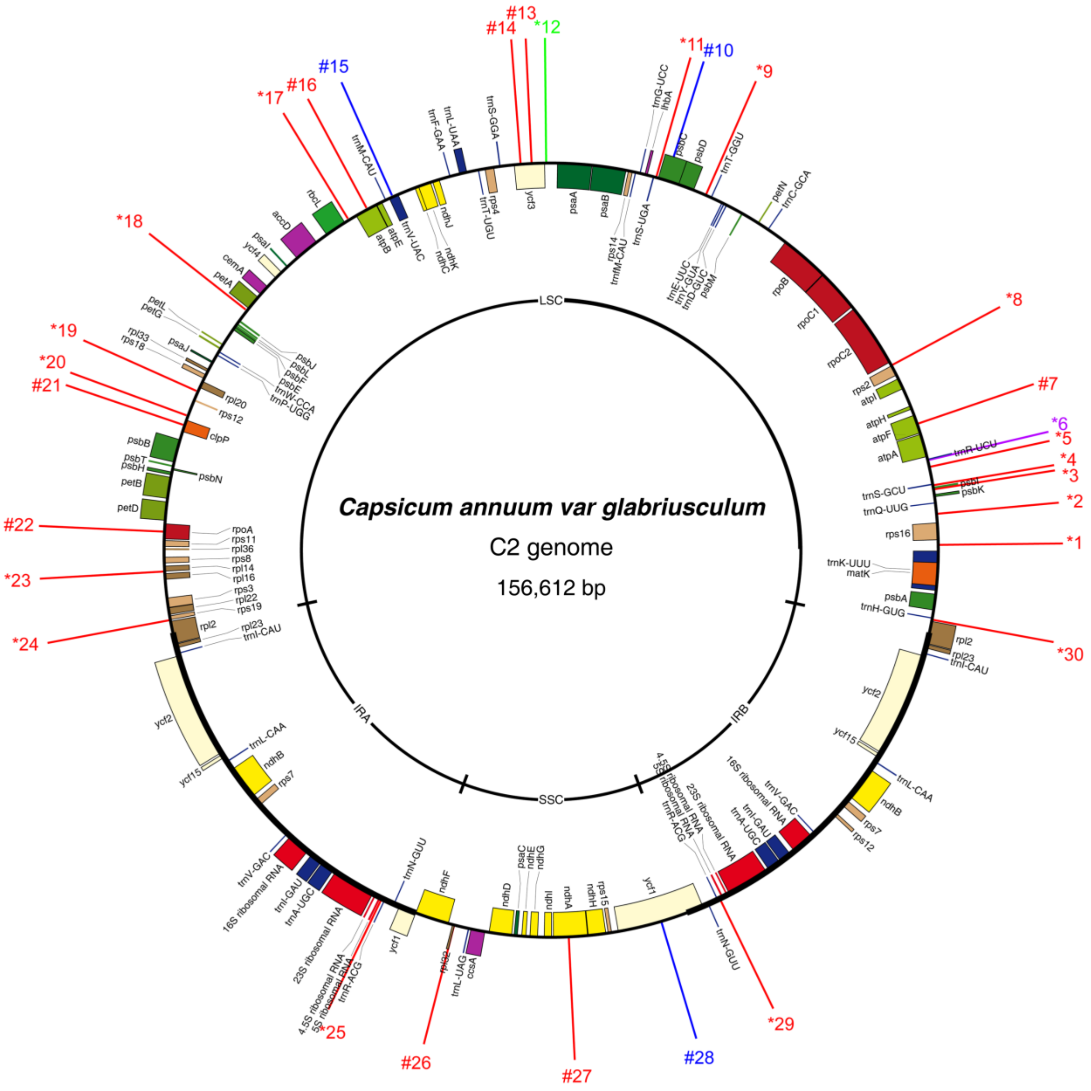

Supplementary Figure S3. Overview of SSR distribution in pepper C2 chloroplast genome.

The C2 genome refers to the chloroplast genome of semi-wild pepper line 'American bird pepper' (*C. annuum var. glabriusculum*). Perfect SSRs with 1, 3 and 4 bp length of motif were represented by red, blue and green lines, respectively. And compound SSRs were showed with purple lines. Number with * and # in the front means that the corresponding SSRs were located in the intergenic and genic region, respectively.

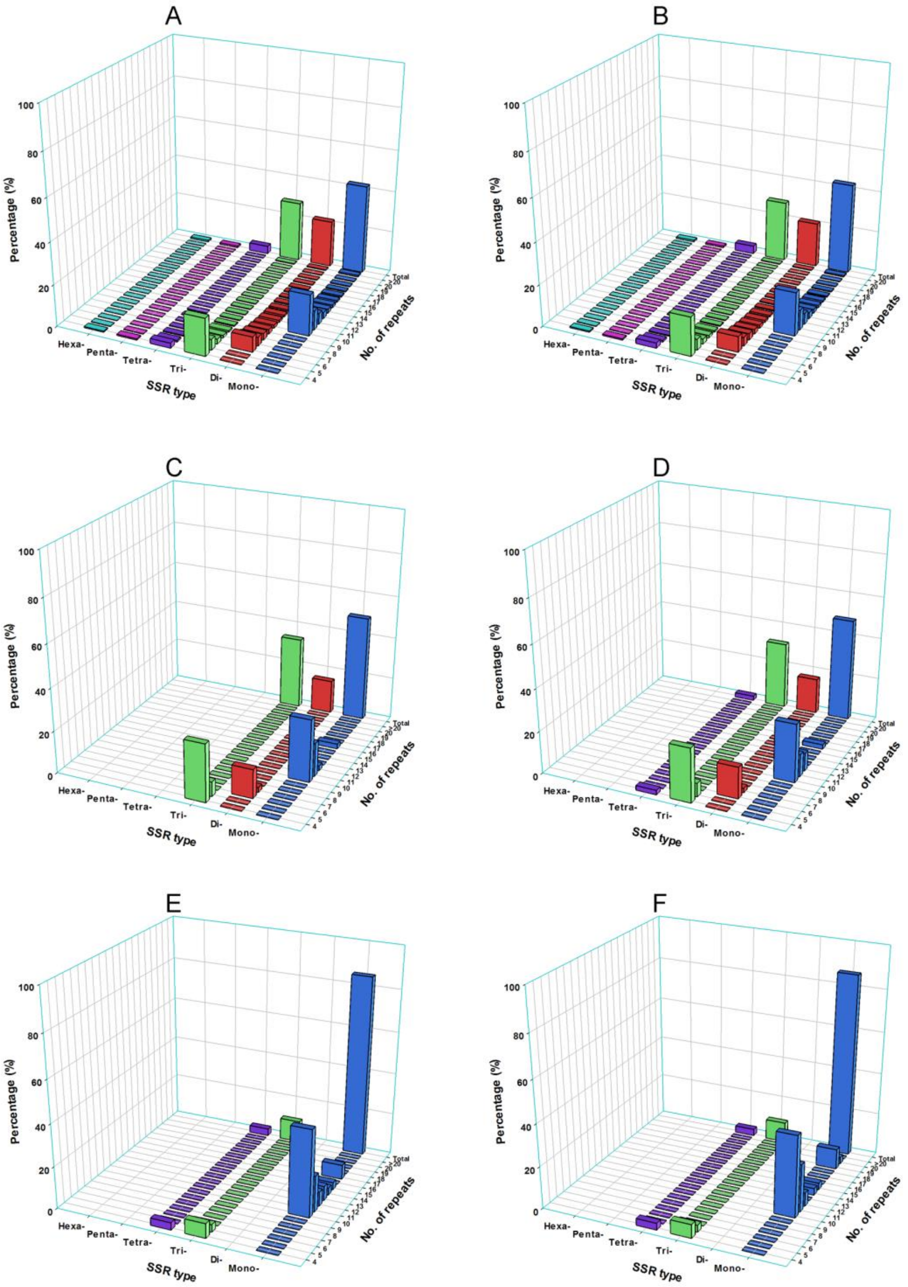

Supplementary Figure S4. Relative frequency of SSR motif with different length, by the number of repeats in six pepper genomes.

A and **B** for the nuclear genomes N1 and N2; **C** and **D** for the mitochondrial genomes M1 and M2; **E** and **F** for the chloroplast genomes C1 and C2, respectively. The graph was based on the total SSR motif identified in nuclear (n_{N1}= 876,580 and n_{N2}= 859,515), mitochondrial (n_{M1}=44 and n_{M2}=47) and chloroplast (n_{C1}=29 and n_{C2}=31) genomes.

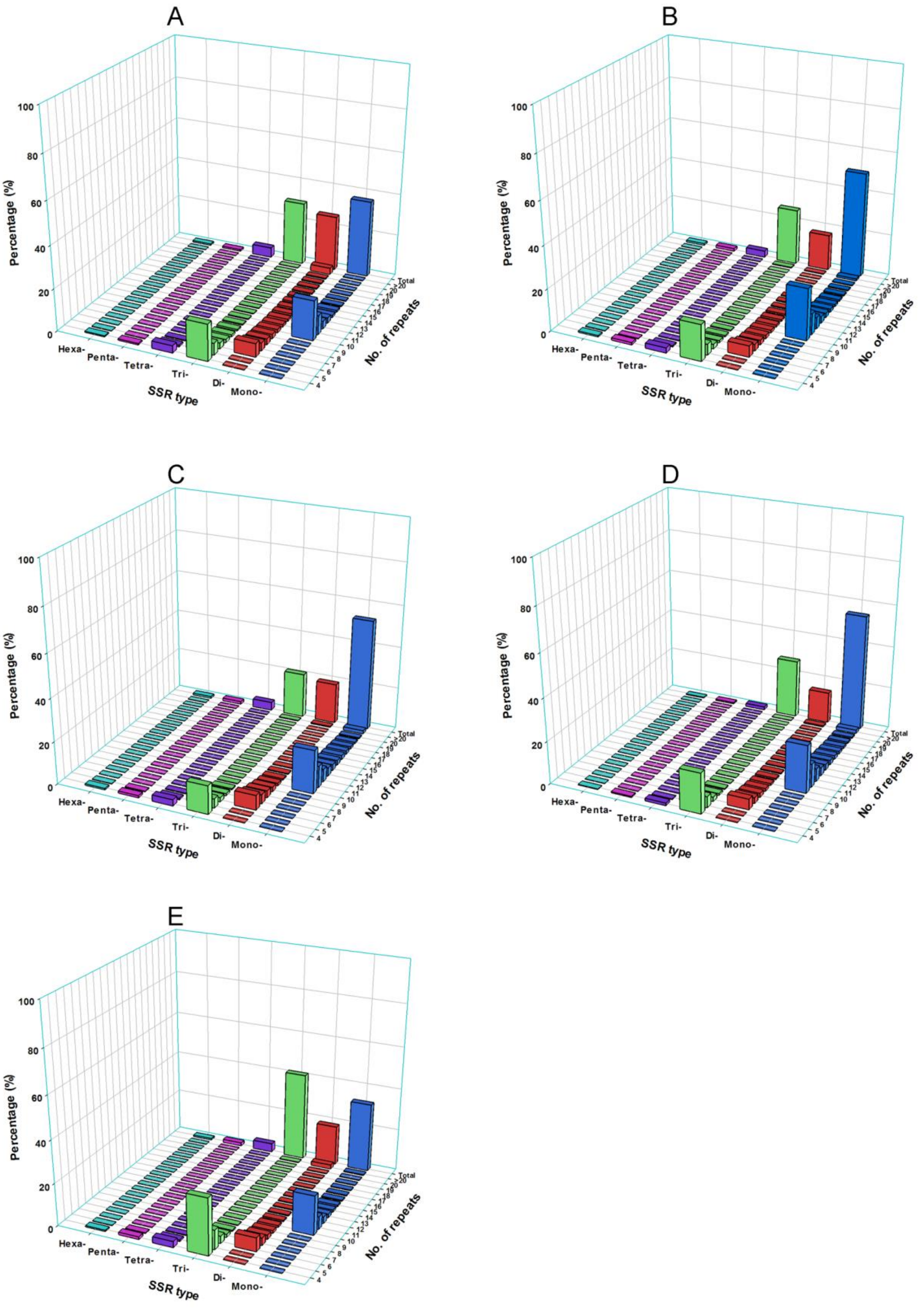

Supplementary Figure S5. Relative frequency of SSR motif with different length, by number of repeats in five plant species.

A, Tomato; **B**, Potato; **C**, Cucumber; **D**, Arabidopsis; **E**, Rice.

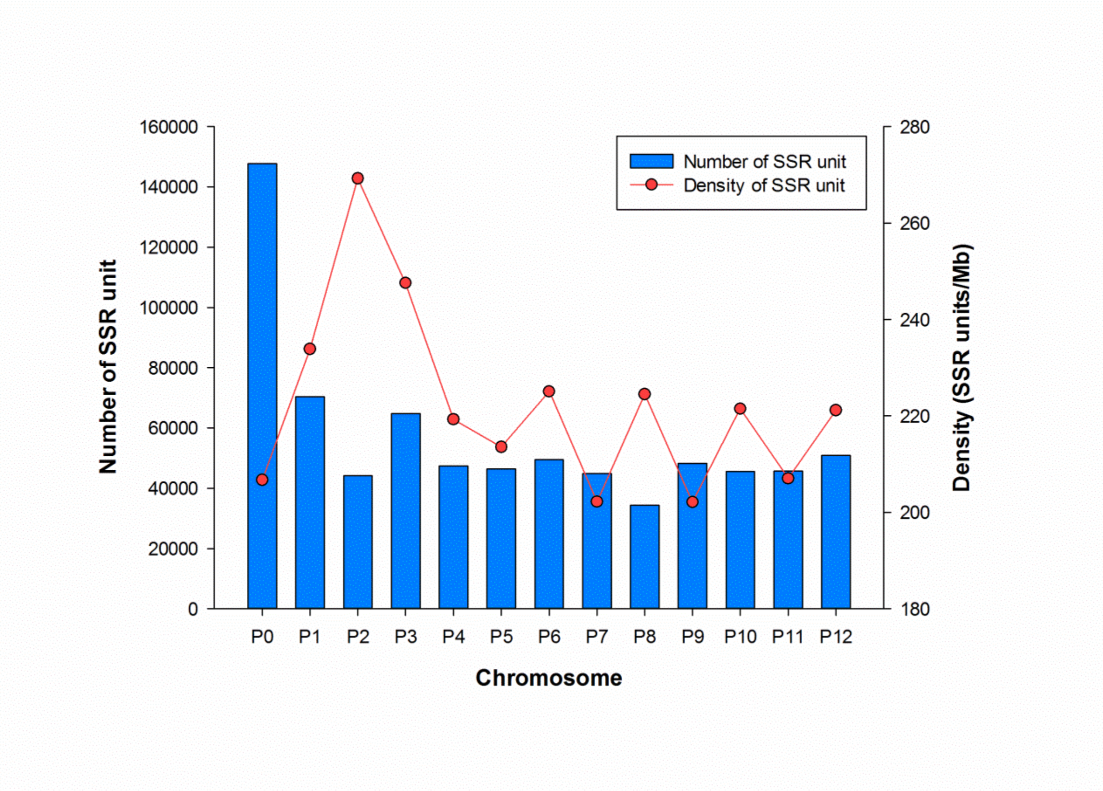

Supplementary Figure S6. Number and density comparison of SSR units on 13 chromosomes of Zunla-1 reference genome.

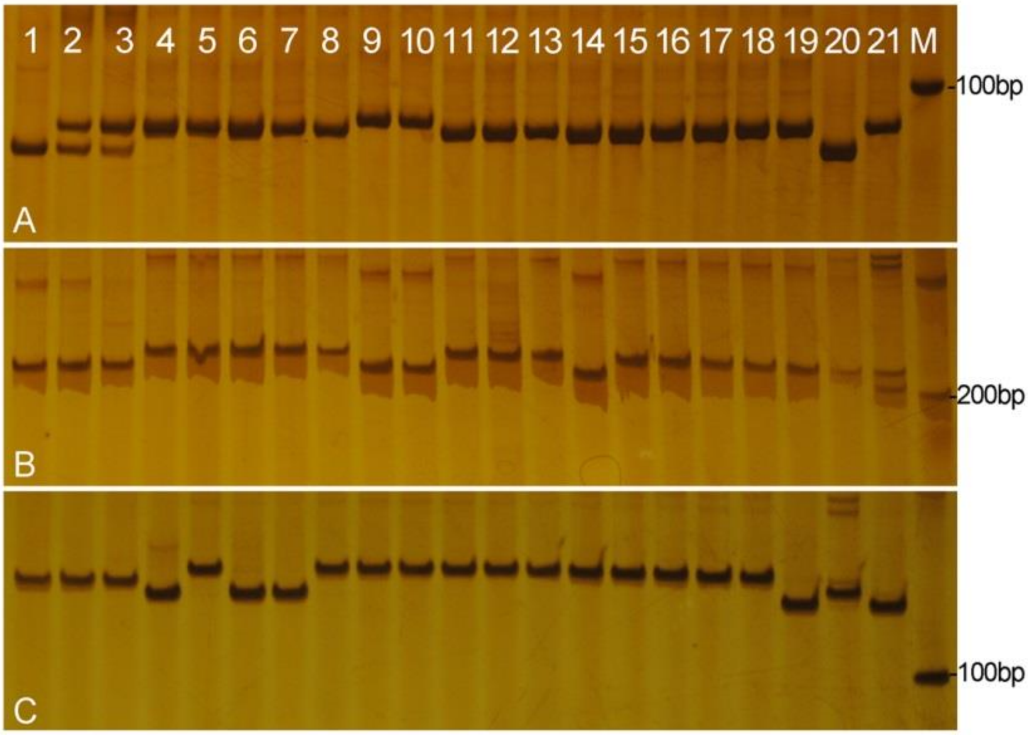

Supplementary Figure S7. Genotyping of 21 pepper lines with three newly developed polymorphic SSR markers.

The order of sampling application on 21 lanes (1~21) was same to the order of samples in **Supplementary Table S1**. Lane M, Marker I (100~600 bp ladder). **A**, **B** and **C** for the marker SSR_P0_580381951, SSR_P0_655489419 and SSR_P0_449855951, respectively.
